# Analysis of Double Elastic Steel Wind Driven Magneto-Electric Vibration Energy Harvesting System

**DOI:** 10.3390/s21217364

**Published:** 2021-11-05

**Authors:** Yi-Ren Wang, Ming-Ching Chu

**Affiliations:** Department of Aerospace Engineering, Tamkang University, Tamsui, New Taipei City 25137, Taiwan; 609430045@gms.tku.edu.tw

**Keywords:** vibration energy harvesting system, piezoelectric patch, nonlinear vibration, frequency response

## Abstract

This research proposes an energy harvesting system that collects the downward airflow from a helicopter or a multi-axis unmanned rotary-wing aircraft and uses this wind force to drive the magnet to rotate, generating repulsive force, which causes the double elastic steel system to slap each other and vibrate periodically in order to generate more electricity than the traditional energy harvesting system. The design concept of the vibration mechanism in this study is to allow the elastic steel carrying the magnet to slap another elastic steel carrying the piezoelectric patch to form a set of double elastic steel vibration energy harvesting (DES VEH) systems. The theoretical DES VEH mechanism of this research is composed of a pair of cantilever beams, with magnets attached to the free end of one beam, and PZT attached to the other beam. This study analyzes the single beam system first. The MOMS method is applied to analyze the frequency response of this nonlinear system theoretically, then combines the piezoelectric patch and the magneto-electric coupling device with this nonlinear elastic beam to analyze the benefits of the system’s converted electrical energy. In the theoretical study of the DES VEH system, the slapping force between the two elastic beams was considered as a concentrated load on each of the beams. Furthermore, both SES and DES VEH systems are studied and correlated. Finally, the experimental data and theoretical results are compared to verify the feasibility and correctness of the theory. It is proven that this DES VEH system can not only obtain the electric energy from the traditional SES VEH system but also obtain the extra electric energy of the steel vibration subjected to the slapping force, which generates optimal power to the greatest extent.

## 1. Introduction

The development of green energy technology has always been an important research topic, and vibration often occurs in life, therefore, the research on vibration energy harvester (VEH) that uses vibration to generate electricity is a very popular field. Roundy et al. [1] proposed the use of micro electromechanical system (MEMS) manufacturing technology to design electro-vibration energy converters. Roundy et al. [2] also found that piezoelectric converters can convert more power than capacitive converters. In addition, piezoelectric converters do not need to provide an additional voltage source, which is one of the reasons why piezoelectric converters are more advantageous than capacitive converters. Roundy and Wright [3] also pointed out that the model of the VEH system based on the principle of piezoelectricity is increasing, and most of them have been verified in practice. Therefore, this technology is not limited to theoretical predictions. In the review work of Iqbal et al. [4], they concluded that due to the large amount of vibration-based energy available in the environment, the VEH has been identified as the energy harvester with the highest potential. Recently, Jiang et al. [5] also reviewed the piezoelectric vibration energy harvesting technologies with magnetic coupling and determine the potential benefits of the magnetic force on energy-harvesting techniques. From this, we can see the rise of mechanical vibration into green energy in the field of energy.

In the aerospace field, Li et al. [6] reviewed the design of using fluid-induced vibration as an energy harvesting device and suggested that nonlinear coupling phenomena should be considered in the design of energy harvesters because these phenomena are the core key to causing vibration. It can be seen from the foregoing that the importance of nonlinear vibration in the VEH system. Nayfeh and Mook [7] proposed that when the natural frequencies of the same degree-of-freedom are multiples, the high-frequency modes are excited but the low-frequency modes produce higher amplitudes. This is a characteristic of typical nonlinearity, which is called internal resonance. They also mentioned a variety of analytical methods for analyzing nonlinear systems, and the method of multiple scales (MOMS) is more conducive to the analysis of non-conservative systems than other methods. Therefore, the MOMS method is very useful for the theoretical analysis of our system.

In the VEH study, Erturk and Inman [8] analyzed the frequency response, the voltage, current, and power output generated by the vibration of the fixed-free Euler–Bernoulli beam with piezoelectric patch. Rajora et al. [9] used Hooke’s Law to calculate the electrical energy conversion performance of piezoelectric patches and learned that piezoelectric patches have different electrical energy conversion benefits in different material thicknesses. Masana and Daqaq [10] applied axial force to a fixed-fixed Euler–Bernoulli beam to generate transversal vibration. In their work, the method of multiple scales (MOMS) was used for analysis. In addition, they also used fixed points plot to analyze the transverse vibration of the beam and proved that the axial disturbance can control the frequency and amplitude of the nonlinear elastic beam in the transverse direction.

Bistable energy harvester (BEH) is another research focus of the non-linear VEH system. Most BEH systems use a cantilever beam as the main mechanism, attach a magnet to its free end, and place a magnet that repels it opposite the free end and use the repulsive force of the magnet to excite the beam. The deformation of the piezoelectric patch is increased, and thus a greater power generation benefit is produced. Andó et al. [11] proposed a 2D BEH, which makes one cantilever beam deflects in the Z direction, and the other cantilever beam deflects in the Y direction. This device can be excited in two different directions at the same time, in order to achieve better power generation. Harne and Wang [12] reviewed the relevant theories about the BEH system and pointed out that the design of the BEH system in various studies largely relies on magnetic attraction, repulsion, and mechanical loading to generate the bistable vibration. Gao et al. [13] connected a spring to a magnet and placed it on an elastic structure and found that the device connected to the spring and the magnet has better electrical energy conversion than traditional BEH. Zhu and Zu [14] designed a magnetic induction buckled beam piezoelectric energy harvester. The beam is fixed at one end, and the other end is movable and subjected to magnetic induction pressure. Their results show that magnetic induction can be used to generate buckled phenomena and can effectively trigger inter-well motion. Wang and Zhu [15] proposed an energy harvesting system with an oscillation horizontal cantilever beam facing the direction of airflow. The effect of airflow velocities on the system’s output power was investigated. Their experimental results show that if the magnet interaction is properly integrated, the power output can be increased by 30%. Wang et al. [16] proposed a rotating magnetic excitation VEH system. They showed that the magnetic force can excite the elastic steel to vibrate nonlinearly to generate electrical energy. These magneto-PZT concepts proved the feasibility of the energy harvesting system. However, only a simplified theoretical model was studied, and the main issue of the slap between steels is not considered, which may enhance the output power.

In addition, in recent years, an energy harvesting system based on the slapping force has been proposed. Fu et al. [17] used three parallel cantilever beams to collide with each other to generate vibration and generated power through the “collision”. They proved that the power generation from the slap between steels is possible. Wang et al. [18] designed two parallel cantilever beams to form a double elastic steel VEH (DES VEH) system. In order to achieve the perfect performance of the slap, the placement of the PZT patch is very important. They obtained the peak and node of the elastic steel of this DES VEH system and placed the PZT at the node to obtain the maximum power generation benefit of this DES VEH system. Wang et al. [19] used the nonlinear model of transverse vibration fixed-fixed beam to simulate the DES VEH system. The position of the PZT and the position of the external force were located to produce the maximum electric power output. The lateral DES VEH model proposed in their research proved to be feasible.

**Figure 1:**
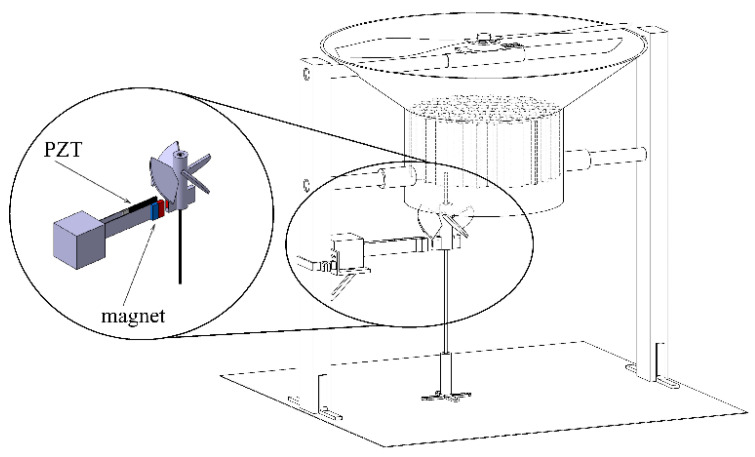
Concept of the wind driven magneto electric vibration energy harvesting system.

In this research, a double elastic steel vibration energy harvester system is presented. This system uses the wind-driven magnetic field to excite the elastic steel to vibrate and generate electricity. This system collects the downward airflow from a helicopter or a multi-axis unmanned rotary-wing aircraft and uses this wind force to drive the magnet to rotate, generating repulsive force, which makes the DES VEH system vibrate periodically. The design concept of the vibration mechanism in this study is to allow the elastic steel carrying the magnet to slap another elastic steel carrying the piezoelectric patch to form a set of DES VEH systems. The results of this study show that this device can not only obtain the electric energy from the traditional energy harvesting system but also obtain the electric energy of the steel vibration subjected to the slapping force. Instead of just considering the deformation of the PZT on a traditional single elastic beam vibration energy harvester, this study implements another characteristic of the PZT electric power generation by subjecting to the clapping force. The present work applies the deformation and mechanical stress on the PZT simultaneously, which perfectly makes the power generation to the greatest extent. The concept of the wind-driven magneto-electric vibration energy harvesting system is shown in Figure 1.

**Figure 2:**
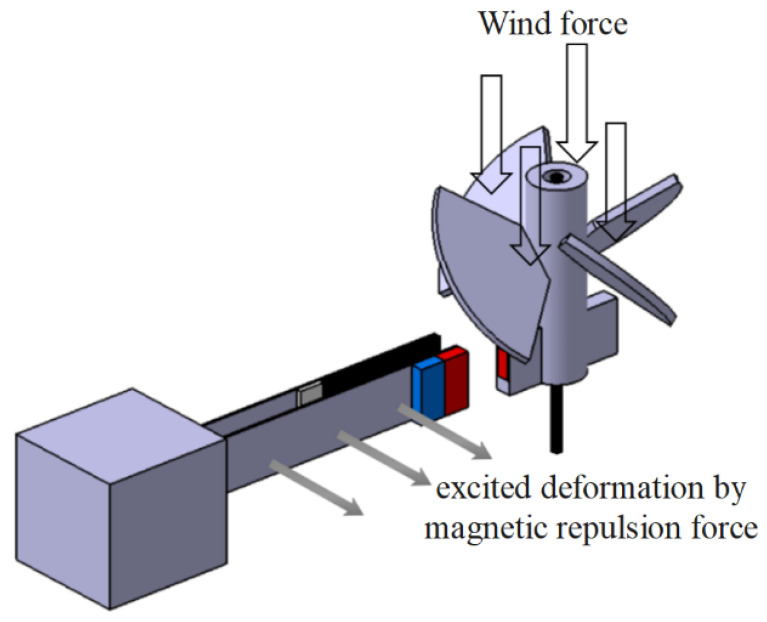
Schematic of the DES VEH.

The theoretical DES VEH mechanism of this research is composed of a pair of cantilever beams, with magnets attached to the free end of one beam, and PZT attached to the other beam as shown in Figure 2. The present work first analyzes the single-beam system. The detailed study of the nonlinear single-beam vibration equation of motion, frequency response, and its power generation benefit are presented in Section 2, Section 3 and Section 4. We obtained the equation of motion of the nonlinear elastic beam by Newton’s 2nd Law, Taylor series expansion, and Euler’s angle transformation. Secondly, we used the MOMS method to analyze the frequency response of this nonlinear system. Then combined the piezoelectric patch and the magneto-electric coupling device with this nonlinear elastic beam to analyze the benefits of the coupling system’s vibration energy converted into electrical energy. The slapping force of the DES VEH system can be considered as a concentrated load on the SES VEH system. A detailed study of the DES VEH system and its power generation benefit are introduced in Section 5. Furthermore, both single elastic steel and double elastic steel VEH systems were studied and correlated. In Section 6, the experiments are performed. The experimental data and theoretical results are compared to verify the feasibility and correctness of the theory and are presented in Section 7. This research also explores the differences between the SES VEH and DES VEH systems in the experiment and analyzes the power generation benefits of the overall nonlinear system.

## 2. Theoretical Model

### 2.1. Beam Equations of Motion

**Figure 3:**
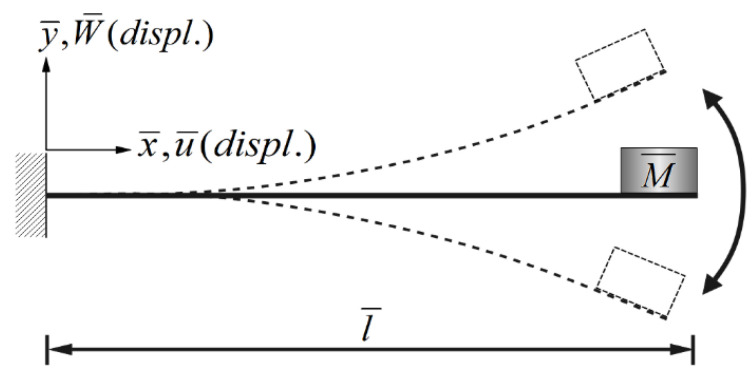
Coordinate of the fixed-free beam with tip mass.

The initial status of the beam was considered straight, and each cross-section was a plane that follows stress-strain laws. Using Newton’s 2nd Law, Euler’s angle transformation, and Taylor series expansion, this study derives the basic equations of motion for the nonlinear beam, but excludes any rotations in the beam; i.e., limiting it to planar motions. The schematic diagram and coordinates of each symbol are shown in Figure 3. According to the nonlinear 2D Euler–Bernoulli beam theory [20], the equations of motion for the 2D beam are as follows:

(1) $$\bar{m}\ddot{\bar{u}}-EA{\bar{u}}^{\prime \prime }=EA(\frac{1}{2}{{\bar{W}}^{\prime }}^{2}-{\bar{u}}^{\prime }{{\bar{W}}^{\prime }}^{2}{)}^{\prime }+EI_{A}({\bar{W}}^{\prime }({\bar{W}}^{\prime \prime \prime }-{\bar{u}}^{\prime \prime \prime }{\bar{W}}^{\prime }-2{\bar{u}}^{\prime \prime }{\bar{W}}^{\prime \prime }-3{\bar{u}}^{\prime }{\bar{W}}^{\prime \prime \prime }){)}^{\prime }$$

(2) $$\begin{array}{l} \bar{m}\ddot{\bar{W}}+EI_{A}{\bar{W}}^{iv}-EA({\bar{u}}^{\prime }{\bar{W}}^{\prime }-{\bar{u}}^{\prime^{2}}{\bar{W}}^{\prime }+\frac{1}{2}{\bar{W}}^{\prime^{3}}{)}^{\prime }+\bar{q}-EI_{A}({\bar{u}}^{\prime }{\bar{W}}^{\prime \prime \prime }+({\bar{u}}^{\prime }{\bar{W}}^{\prime }{)}^{\prime \prime }\\ -({\bar{u}}^{\prime^{2}}-{\bar{W}}^{\prime^{2}}){\bar{W}}^{\prime \prime \prime }-{\bar{u}}^{\prime }({\bar{u}}^{\prime }{\bar{W}}^{\prime }{)}^{\prime \prime }-({\bar{u}}^{\prime^{2}}{\bar{W}}^{\prime }-\frac{1}{3}{\bar{W}}^{\prime^{3}}{)}^{\prime \prime }{)}^{\prime }=0 \end{array}$$

In Equations (1) and (2), $\bar{m}$ is the mass of the beam, *E* is Young’s modulus, *A* represents the cross-section area of the beam, *I_A_* is the moment of inertia of the beam, $\bar{q}$ denotes the uniform distributed force on the beam, ${\left(\right)}^{\prime }$ is ($d/d\bar{x}$), $\overset{\cdot }{\left(\right)}$ represents ($d/d\bar{t}$), and the $\bar{u}$ and $\bar{W}$ represent the displacements on $\bar{x}$ and $\bar{y}$ directions, respectively. $\bar{M}$ is the tip mass (of the magnet). It is noted that the magnet mass is treated as the boundary condition of the beam-free end and is not shown in the equation of motion of the beam. The boundary conditions are:

(3) $$\left\{ \begin{array}{l} \bar{W}(0,\bar{t})=0\\ {\bar{W}}^{\prime }(0,\bar{t})=0\\ EI_{A}{\bar{W}}^{\prime \prime }(\bar{l},\bar{t})=I_{m}{\bar{\omega }}^{2}{\bar{W}}^{\prime }(\bar{l},\bar{t})\\ EI_{A}{\bar{W}}^{\prime \prime \prime }(\bar{l},\bar{t})=-\bar{M}{\bar{\omega }}^{2}\bar{W}(\bar{l},\bar{t}) \end{array}\right.$$

where $\bar{\omega }$ denotes $\sqrt{EI_{A}/\bar{m}{\bar{l}}^{4}}$.

The axial force on the free end in the $\bar{x}$ direction is zero, therefore, this equation of motion can be simplified to a simple $\bar{y}$ direction equation, which is expressed as follows:

(4) $$\begin{array}{l} \bar{m}\ddot{\bar{W}}+\bar{\mu }\dot{\bar{W}}+EI_{A}({\bar{W}}^{\prime \prime \prime }+{\bar{W}}^{\prime }{\bar{W}}^{{\prime \prime }^{2}}+{\bar{W}}^{\prime \prime \prime }{\bar{W}}^{\prime^{2}}{)}^{\prime }\\ =-\frac{1}{2}\bar{m}({\bar{W}}^{\prime }\int_{\bar{l}}^{\bar{x}}(\int_{0}^{\bar{x}}{\bar{W}}^{\prime^{2}}d\bar{x})d\bar{x}{)}^{\prime }+\bar{q} \end{array}$$

It is noted that we add a damping term in Equation (4), where $\bar{\mu }$ denotes the damping coefficient of the beam. The dimensionless equation of Equation (4) can be expressed as follows:

(5) $$W^{**}+\mu W^{*}+W^{iv}+{W^{\prime \prime }}^{3}+W^{iv}{W^{\prime }}^{2}+4W^{\prime }W^{\prime \prime \prime }W^{\prime \prime }=-\frac{1}{2}(W^{\prime }\int_{1}^{x}{\left(\int_{0}^{x}{W^{\prime }}^{2}dx\right)}^{**}dx{)}^{\prime }+q$$

where $l=\frac{\bar{l}}{\bar{l}}=1$, $\tau =\bar{\omega }\bar{t}$, $W=\frac{\bar{W}}{\bar{l}}$, $\tau =\sqrt{\frac{EI}{\bar{m}{\bar{l}}^{4}}}\bar{t}$, $\mu =\frac{{\bar{l}}^{2}}{\sqrt{\bar{m}EI_{A}}}\bar{\mu }$, $q=\frac{\bar{q}}{\bar{m}\bar{l}{\bar{\omega }}^{2}}$, $\bar{\omega }=\sqrt{\frac{EI_{A}}{\bar{m}{\bar{l}}^{4}}}$, ${\left(\right)}^{\prime }$ represents d/dx, ${\left(\right)}^{*}$ denotes d/dτ.

The dimensionless boundary conditions are expressed as follows:

(6) $$\left\{ \begin{array}{l} W(0,\tau )=0\;,\\ W^{\prime }(0,\tau )=0,\\ W^{\prime \prime }(l,\tau )=0,\;\\ W^{\prime \prime \prime }(l,\tau )=-MW(l,\tau ) \end{array}\right.$$

where $M=\frac{\bar{M}}{\bar{l}\bar{m}}$.

### 2.2. Theoretical Model of Piezoelectric Patch

This study uses the piezoelectric equation of the piezoelectric patch (PZT patch) of Rajora et al. [9] as follows:

(7) $$C_{p}\dot{V}+\frac{1}{{\bar{R}}_{p}}V+\int_{\bar{a}}^{\bar{b}}eh_{p}t_{h}{\dot{\bar{W}}}^{\prime \prime }d\bar{x}=0$$

where ${\bar{R}}_{P}$ is resistance, *e* is the piezoelectric coupling coefficient, *h_p_* denotes the thickness of the piezo patch, *t_h_* represents the width of the piezo patch, *C_p_* is the capacitance of the piezoelectric patch. The Coulomb force of this PZT patch acting on the nonlinear elastic beam is:

(8) $$\mathrm{Coulomb}\;\mathrm{force}=\int_{\bar{a}}^{\bar{b}}eh_{p}t_{h}{\dot{\bar{W}}}^{\prime \prime }d\bar{x}\frac{V}{h_{p}}=(et_{h}\int_{\bar{a}}^{\bar{b}}{\bar{W}}^{\prime \prime }d\bar{x})V=C_{f}(\int_{\bar{a}}^{\bar{b}}{\bar{W}}^{\prime \prime }d\bar{x})V$$

where $\bar{a}$ and $\bar{b}$ are the positions of the piezo patch from one end to the other end, $C_{f}$ is the Piezoelectric coupling coefficient, The dimensionless PZT equation can be written as:

(9) $$\nu^{*}+R_{p}\nu +\hat{k}\int_{a}^{b}{\left(W^{\prime \prime }\right)}^{*}dx=0$$

where $v=\frac{V}{C_{f}}$, $R_{P}=\frac{1}{{\bar{R}}_{p}C_{p}\bar{\omega }}$, $\hat{k}=\frac{eh_{p}t_{h}}{C_{p}C_{f}}$, ${\left(\right)}^{*}=d/d\tau$, ${\left(\right)}^{\prime }=d/dx$. The dimensionless voltage (v) can be found from Equation (9):

(10) $$\nu =-\frac{\hat{k}}{e^{R_{P}\tau }}\int_{0}^{\tau }(\int_{a}^{b}{\left(W^{\prime \prime }\right)}^{*}dx)e^{R_{P}\tau }d\tau$$

From Equation (10), we can obtain the dimensionless Coulomb force of the PZT acting on the nonlinear beam. Let $\eta^{2}=\frac{C_{f}^{2}}{\bar{l}\bar{m}{\bar{\omega }}^{2}}$, we can obtain:

(11) $$\frac{C_{f}^{2}(\int_{a}^{b}W^{\prime \prime }dx)\nu }{\bar{l}\bar{m}{\bar{\omega }}^{2}}=\eta^{2}(\int_{a}^{b}W^{\prime \prime }dx)\nu =-\frac{\hat{k}\eta^{2}}{e^{R_{P}\tau }}\int_{a}^{b}W^{\prime \prime }dx\int_{0}^{\tau }(\int_{a}^{b}{\left(W^{\prime \prime }\right)}^{*}dx)e^{R_{P}\tau }d\tau$$

We combine the PZT force (Equation (11)) with Equation (5) to obtain the dimensionless beam equation with PZT as:

(12) $$\begin{array}{l} W^{**}+W^{iv}+\mu W^{*}+{W^{\prime \prime }}^{3}+W^{iv}{W^{\prime }}^{2}+4W^{\prime }W^{\prime \prime \prime }W^{\prime \prime }\\ -\frac{\hat{k}\eta^{2}}{e^{R_{P}\tau }}\int_{a}^{b}W^{\prime \prime }dx\int_{0}^{\tau }(\int_{a}^{b}{\left(W^{\prime \prime }\right)}^{*}dx)e^{R_{P}\tau }d\tau =-\frac{1}{2}(W^{\prime }\int_{1}^{x}{\left(\int_{0}^{x}{W^{\prime }}^{2}dx\right)}^{**}dx{)}^{\prime }+q \end{array}$$

### 2.3. Theoretical Model of Magneto-Electric Equation

The external force exerted by the magnet’s magneto-electric effect on the nonlinear elastic beam can be expressed by Lorentz Force. The magnet’s magneto-electric equation is:

(13) $${\bar{C}}_{M}\dot{\bar{I}}+{\bar{R}}_{M}\bar{I}+{\bar{C}}_{G}\dot{\bar{W}}=0$$

where ${\bar{C}}_{G}$ is the electromagnetic coupling coefficient, ${\bar{C}}_{M}$ is inductance, $\bar{I}$ is current, ${\bar{R}}_{M}$ is resistance. The magneto-electric Lorentz force acting on the nonlinear elastic beam is:

(14) $$\mathrm{Lorentz}\;\mathrm{force}\;={\bar{C}}_{G}\bar{I}=B\bar{L}\sin\theta$$

where *B* is the magnetic field and $\bar{L}$ is the length of the beam. The dimensionless equation is:

(15) $$i^{*}+R_{M}i+W^{*}=0$$

The dimensionless current is:

(16) $$i=-\frac{1}{e^{R_{M}\tau }}\int_{0}^{\tau }W^{*}e^{R_{M}\tau }d\tau$$

where $R_{M}=\frac{{\bar{R}}_{M}}{{\bar{C}}_{M}\bar{\omega }}$. Substituting Equation (16) into Equation (12), the equation of motion integrating magnetoelectricity and piezoelectricity can be obtained as follows:

(17) $$\begin{array}{l} W^{**}+W^{iv}+\mu W^{*}+{W^{\prime \prime }}^{3}+W^{iv}{W^{\prime }}^{2}+4W^{\prime }W^{\prime \prime \prime }W^{\prime \prime }\\ -\frac{\hat{k}\eta^{2}}{e^{R_{P}\tau }}\int_{a}^{b}W^{\prime \prime }dx\int_{0}^{\tau }{\left(\int_{a}^{b}{\left(W^{\prime \prime }\right)}^{*}dx\right)}^{}e^{R_{p}\tau }d\tau +C_{G}\frac{1}{e^{R_{M}\tau }}\int_{0}^{\tau }W^{*}e^{R_{M}\tau }d\tau \\ =-\frac{1}{2}(W^{\prime }\int_{1}^{x}{\left(\int_{0}^{x}{W^{\prime }}^{2}dx\right)}^{**}dx{)}^{\prime }+q \end{array}$$

where $C_{G}=\frac{{\bar{C}}_{G}^{2}}{{\bar{C}}_{M}\bar{m}{\bar{\omega }}^{2}}$.

### 2.4. Method of Multiple Scales, MOMS

We use the method of multiple scales (MOMS) of Nayfeh and Mook [7] to analyze this nonlinear problem. The MOMS method divides time into two time scales, namely, the fast and the slow time scales. Let the $T_{0}=\tau$ be the fast-time term and $T_{1}=\varepsilon^{2}\tau$ be the slow-time term and $W(x,\tau ,\varepsilon )=\varepsilon^{1}W_{0}(T_{0},T_{1},T_{2},\ldots .)+\varepsilon^{3}W_{1}(T_{0},T_{1},T_{2},\ldots .)$. Equation (17) can be expressed as:

(18) $$\begin{array}{l} \left(\varepsilon \frac{\partial^{2}W_{0}}{\partial T_{0}{}^{2}}+\varepsilon^{3\;}\;\frac{\partial^{2}W_{1}}{\partial T_{0}{}^{2}}+2\varepsilon^{3\;}\frac{\partial^{2}W_{0}}{\partial T_{0}\partial T_{1}}+\cdots )+(\varepsilon W_{0}{}^{iv}+\varepsilon^{3}W_{1}{}^{iv}\right)\\ +\varepsilon^{2}\mu (\varepsilon \frac{\partial W_{0}}{\partial T_{0}}+\varepsilon^{3}\frac{\partial W_{1}}{\partial T_{0}}+\varepsilon^{3}\frac{\partial W_{0}}{\partial T_{1}}+\cdots )+(\varepsilon^{3}{W^{\prime \prime }}_{0}{}^{\;3}+2\varepsilon^{5}{W^{\prime \prime }}_{0}{}^{\;2}{W^{\prime \prime }}_{1}+\cdots )\\ +(\varepsilon^{3}W_{0}^{\mathrm{iv}}{W^{\prime }}_{0}{}^{\;2}+2\varepsilon^{5}W_{0}^{\mathrm{iv}}{W^{\prime }}_{0}{W^{\prime }}_{1}+\varepsilon^{5}W_{1}^{iv}{W^{\prime }}_{0}^{2}+\cdots )\\ +4(\varepsilon^{3}{W^{\prime }}_{0}\;{W^{\prime \prime \prime }}_{0}\;{W^{\prime \prime }}_{0}+\varepsilon^{5}{W^{\prime }}_{0}\;{W^{\prime \prime \prime }}_{0}\;{W^{\prime \prime }}_{1}+\varepsilon^{5}{W^{\prime }}_{0}\;{W^{\prime \prime \prime }}_{1}\;{W^{\prime \prime }}_{0}+\varepsilon^{5}{W^{\prime }}_{1}\;{W^{\prime \prime \prime }}_{0}\;{W^{\prime \prime }}_{0}+\cdots )\\ -\frac{\hat{k}\eta^{2}}{e^{R\tau }}\int_{a}^{b}\left(\varepsilon {W^{\prime \prime }}_{0}+\varepsilon^{3}{W^{\prime \prime }}_{1}\right)dx\int_{0}^{\tau }\left(\int_{a}^{b}(\frac{\partial^{3}(\varepsilon {W^{\prime \prime }}_{0}+\varepsilon^{3}{W^{\prime \prime }}_{1})}{\partial T_{0}\partial x^{2}})dxe^{R\tau }\right)d\tau \\ +C_{G}\frac{1}{e^{R\tau }}\int_{0}^{\tau }(\varepsilon \frac{\partial W_{0}}{\partial T_{0}}+\varepsilon^{3}\frac{\partial W_{1}}{\partial T_{0}}+\varepsilon^{3}\frac{\partial W_{0}}{\partial T_{1}})e^{R\tau }d\tau \\ =-\frac{1}{2}\varepsilon^{3}({W^{\prime }}_{0}\int_{1}^{x}{\left(\int_{0}^{x}{W^{\prime }}_{0}{}^{2}dx\right)}^{**}dx{)}^{\prime }+\varepsilon^{3}q \end{array}$$

where ε is the time scale of small disturbances and is a minimum value, the effect of high order terms $\varepsilon^{5},\varepsilon^{7}\ldots$ on the system is neglected. The term composed of the order of $\varepsilon^{1}$ in Equation (18) is:

(19) $$\frac{\partial^{2}W_{0}}{\partial T_{0}{}^{2}}+W_{0}{}^{iv}=0$$

The term composed of the order of $\varepsilon^{3}$ is:

(20) $$\begin{array}{l} \;\frac{\partial^{2}W_{1}}{\partial T_{0}{}^{2}}+W_{1}{}^{iv}=-2\;\frac{\partial^{2}W_{0}}{\partial T_{0}\partial T_{1}}-\mu \frac{\partial W_{0}}{\partial T_{0}}-{W^{\prime \prime }}_{0}{}^{\;3}-W_{0}^{\mathrm{iv}}{W^{\prime }}_{0}{}^{\;2}-4{W^{\prime }}_{0}\;{W^{\prime \prime \prime }}_{0}\;{W^{\prime \prime }}_{0}\;\\ -\frac{1}{2}({W^{\prime }}_{0}\int_{1}^{x}{\left(\int_{0}^{x}{W^{\prime }}_{0}{}^{2}dx\right)}^{**}dx{)}^{\prime }-C_{G}\frac{1}{e^{R\tau }}\int_{0}^{\tau }(\frac{\partial W_{0}}{\partial T_{0}})e^{R\tau }d\tau \\ +\frac{\hat{k}\eta^{2}}{e^{R\tau }}\int_{a}^{b}{W^{\prime \prime }}_{0}dx\int_{0}^{\tau }(\int_{a}^{b}(\frac{\partial^{3}W_{0}}{\partial T_{0}\partial x^{2}})dxe^{R\tau })d\tau +q \end{array}$$

## 3. Frequency Response

### 3.1. Eigen Value and Frequency

The *W*_0_ is defined as *W*_0_ = *X*(*x*)*Y*(*t*) by using the separation of variables. Equation (19) can be written as:

(21) $$X(x)\ddot{Y}(\tau )+X^{iv}(x)Y(\tau )=0$$

With the boundary conditions, the characteristic equation can be found as:

(22) $$\alpha^{3}(-1-\cos\alpha l\cosh\alpha l)+M(-\sin\alpha l\cosh\alpha l+\cos\alpha l\sinh\alpha l)=0$$

The mode shape of the *n*th harmonic is:

(23) $$\phi_{n}(x)=(-\sin\alpha_{n}x-\sinh\alpha_{n}x)+\frac{\left(\sin\alpha_{n}+\sinh\alpha_{n}\right)}{\left(-\cos\alpha_{n}-\cosh\alpha_{n}\right)}(-\cos\alpha_{n}x-\cosh\alpha_{n}x)$$

The general solution of *W*_0_ can be expressed as:

(24) $$W_{0}(x,\tau )=\sum_{n=1}^{\infty }\xi_{0n}(\tau )\phi_{n}(x)$$

In which $\xi_{0n}(\tau )$ is expressed as:

(25) $$\xi_{0n}(\tau )=B_{n}(T_{1})e^{-i\zeta_{n}}e^{i\omega_{n}T_{0}}+{\bar{B}}_{n}{(T}_{1})e^{i\zeta_{n}}e^{-i\omega_{n}T_{0}}$$

where $\zeta_{n}$ is phase angle, *B_n_* represents the amplitude of the *n*th mode.

### 3.2. System Frequency Response

Equations (19) and (20) are multiplied by $\phi_{m}$ using the mode shapes’ orthogonal properties, integrated by 0–ℓ (dimensionless ℓ=1). The integral value is zero when $\phi_{m}\neq \phi_{n}$ and is not zero when $\phi_{m}=\phi_{n}$. The equations, based on the order of $\varepsilon^{1}$, can be expressed as follows:

(26) $$\xi_{0m}^{**}+\alpha_{m}^{4}\xi_{0m}=0$$

Order of $\varepsilon^{3}$:

(27) $$\begin{array}{l} \xi_{1m}^{**}+\alpha_{m}^{4}\xi_{1m}=-2\frac{\partial }{\partial T_{1}}\xi_{0m}^{*}-\mu \xi_{0m}^{*}\\ -\frac{1}{Q_{m}}\xi_{0i}\xi_{0j}\xi_{0k}(\int_{0}^{1}{\phi^{\prime \prime }}_{i}{\phi^{\prime \prime }}_{j}{\phi^{\prime \prime }}_{k}\phi_{m}dx+\int_{0}^{1}\alpha_{m}^{4}\phi_{m}{\phi^{\prime }}_{j}{\phi^{\prime }}_{k}\phi_{m}dx+4\int_{0}^{1}{\phi^{\prime }}_{i}{\phi^{\prime \prime }}_{j}{\phi^{\prime \prime \prime }}_{k}\phi_{m}dx)\\ -\frac{1}{2Q_{m}}\xi_{0i}(\xi_{0j}^{**}\xi_{0k}+2\xi_{0j}^{*}\xi_{0k}^{*}+\xi_{0j}\xi_{0k}^{**})(\int_{0}^{1}\phi_{m}({\phi^{\prime \prime }}_{i}\int_{1}^{x}\int_{0}^{x}{\phi^{\prime }}_{j}{\phi^{\prime }}_{k}dxdx+{\phi^{\prime }}_{i}(\int_{1}^{x}\int_{0}^{x}{\phi^{\prime }}_{j}{\phi^{\prime }}_{k}dxdx{)}^{\prime })dx)\\ +\frac{1}{Q_{m}}\frac{k{\hat{\eta }}^{2}}{e^{R\tau }}\int_{0}^{1}\phi_{m}(\int_{a}^{b}{\phi^{\prime \prime }}_{i}\xi_{0}{}_{i}dx\int_{0}^{\tau }\left(\int_{a}^{b}{\phi^{\prime \prime }}_{j}\xi_{0j}^{*}dx\right)e^{R\tau }d\tau )dx-C_{G}\frac{1}{e^{R\tau }}\int_{0}^{\tau }\xi_{om}^{*}e^{R\tau }d\tau +q_{m} \end{array}$$

where $Q_{m}=\int_{0}^{l}\phi_{m}^{2}dx$. In order to consider the frequency response of the system, we assume that the system is subjected to a simple harmonic external force as:

(28) $$q_{m}={\overset{⌢}{q}}_{m}e^{i\Omega T_{0}}={\overset{⌢}{q}}_{m}e^{i(\omega_{m}+\varepsilon^{2}\sigma )T_{0}}={\overset{⌢}{q}}_{m}e^{i\omega_{m}T_{0}}e^{i\sigma T_{1}}$$

where $\Omega =\omega_{m}+\varepsilon \sigma$, σ denotes the tuned frequency near the linear natural frequency ($\omega_{m}$). In order to find the response of the 1st mode (*m* = 1), the secular terms need to select harmonic with the $\omega_{1}$ term. Substituting Equations (25) and (28) into Equation (27), and exciting the 1st mode (*m* = 1), the secular terms of Equation (27) are:

(29) $$\begin{array}{l} -2(i\omega_{1}B_{1}e^{i\zeta_{1}}e^{i\omega_{1}T_{0}}-\omega_{1}\varsigma_{1}^{*}B_{1}e^{-i\zeta_{1}}e^{i\omega_{1}T_{0}})-\mu (i\omega_{1}B_{1}e^{-i\varsigma_{1}}e^{i\omega_{1}T_{0}})+{\overset{⌢}{q}}_{1}e^{i\omega_{1}T_{0}}e^{i\sigma T_{1}}\\ -\frac{1}{Q_{1}}(3B_{1}\bar{B_{1}}B_{1}e^{-i\varsigma_{1}}e^{i\omega_{1}T_{0}}(\int_{0}^{1}\phi_{1}{\phi^{\prime \prime }}_{1}^{3}dx+\int_{0}^{1}\phi_{1}\phi_{1}^{iv}{\phi^{\prime }}_{1}{}^{2}dx+\int_{0}^{1}4\phi_{1}{\phi^{\prime }}_{1}{\phi^{\prime \prime }}_{1}{\phi^{\prime \prime \prime }}_{1}dx)\\ +2B_{1}\bar{B_{2}}B_{2}e^{-i\varsigma_{1}}e^{i\omega_{1}T_{0}}(\int_{0}^{1}3{\phi^{\prime \prime }}_{1}{\phi^{\prime \prime }}_{2}^{2}\phi_{1}dx+\int_{0}^{1}\phi_{1}\phi_{1}^{iv}{\phi^{\prime }}_{2}{}^{2}dx+\int_{0}^{1}2\phi_{1}\phi_{2}^{iv}{\phi^{\prime }}_{1}{\phi^{\prime }}_{2}dx\\ +\int_{0}^{1}4{\phi^{\prime }}_{1}{\phi^{\prime \prime }}_{2}{\phi^{\prime \prime \prime }}_{2}\phi_{1}dx+\int_{0}^{1}4{\phi^{\prime }}_{2}{\phi^{\prime \prime }}_{2}{\phi^{\prime \prime \prime }}_{1}\phi_{1}dx+\int_{0}^{1}4{\phi^{\prime }}_{2}{\phi^{\prime \prime }}_{1}{\phi^{\prime \prime \prime }}_{2}\phi_{1}dx)\\ -2B_{1}\bar{B_{3}}B_{3}e^{-i\varsigma_{1}}e^{i\omega_{1}T_{0}}(\int_{0}^{1}3{\phi^{\prime \prime }}_{1}{\phi^{\prime \prime }}_{3}^{2}\phi_{1}dx+\int_{0}^{1}\phi_{1}^{iv}\phi_{1}{\phi^{\prime }}_{3}{}^{2}dx+\int_{0}^{1}2\phi_{1}\phi_{3}^{iv}{\phi^{\prime }}_{1}{\phi^{\prime }}_{3}dx\\ +\int_{0}^{1}4{\phi^{\prime }}_{1}{\phi^{\prime \prime }}_{3}{\phi^{\prime \prime \prime }}_{3}\phi_{1}dx+\int_{0}^{1}4{\phi^{\prime }}_{3}{\phi^{\prime \prime }}_{3}{\phi^{\prime \prime \prime }}_{1}\phi_{1}dx+\int_{0}^{1}4{\phi^{\prime }}_{3}{\phi^{\prime \prime }}_{1}{\phi^{\prime \prime \prime }}_{3}\phi_{1}dx))\\ -\frac{1}{2Q_{1}}(-4\omega_{1}^{2}B_{1}\bar{B_{1}}B_{1}e^{-i\varsigma_{1}}e^{i\omega_{1}T_{0}}(\int_{0}^{1}{\phi^{\prime \prime }}_{1}\phi_{1}(\int_{1}^{x}\int_{0}^{x}{\phi^{\prime }}_{1}{\phi^{\prime }}_{1}dxdx)dx\\ +\int_{0}^{1}{\phi^{\prime }}_{1}\phi_{1}(\int_{1}^{x}\int_{0}^{x}{\phi^{\prime }}_{1}{\phi^{\prime }}_{1}dxdx{)}^{\prime }dx)\\ +4(-\omega_{1}^{2}-\omega_{2}^{2})B_{1}\bar{B_{2}}B_{2}e^{-i\varsigma_{1}}e^{i\omega_{1}T_{0}}(\int_{0}^{1}{\phi^{\prime \prime }}_{2}\phi_{1}(\int_{1}^{x}\int_{0}^{x}{\phi^{\prime }}_{1}{\phi^{\prime }}_{2}dxdx)dx\\ +\int_{0}^{1}{\phi^{\prime }}_{2}\phi_{1}(\int_{1}^{x}\int_{0}^{x}{\phi^{\prime }}_{1}{\phi^{\prime }}_{2}dxdx{)}^{\prime }dx)\\ +4(-\omega_{1}^{2}-\omega_{3}^{2})B_{1}\bar{B_{3}}B_{3}e^{-i\varsigma_{1}}e^{i\omega_{1}T_{0}}(\int_{0}^{1}{\phi^{\prime \prime }}_{3}\phi_{1}(\int_{1}^{x}\int_{0}^{x}{\phi^{\prime }}_{1}{\phi^{\prime }}_{3}dxdx)dx\\ +\int_{0}^{1}{\phi^{\prime }}_{3}\phi_{1}(\int_{1}^{x}\int_{0}^{x}{\phi^{\prime }}_{1}{\phi^{\prime }}_{3}dxdx{)}^{\prime }dx))-C_{G}\frac{1}{e^{R\tau }}\int_{0}^{\tau }(i\omega_{1})(B_{1}e^{-i\zeta_{1}}e^{i\omega_{1}T_{0}})e^{R\tau }d\tau \\ -C_{G}\frac{1}{e^{R\tau }}\int_{0}^{\tau }(i\omega_{1})(B_{1}e^{-i\zeta_{1}}e^{i\omega_{1}T_{0}})e^{R\tau }d\tau \end{array}$$

where $Q_{1}=\int_{0}^{l}\phi_{1}^{2}dx$. Similarly, for the 2nd mode, the secular terms need to select harmonic with the $\omega_{2}$ term. For the 3rd mode, the secular terms need to select harmonic with the $\omega_{3}$ term, and these secular terms will no longer be listed for the sake of simplicity. After the secular terms are selected, they are determined to be zero; therefore, a solvability condition can be obtained.

Taking the external disturbance of the first mode as an example, the solvability condition of the 1st mode is multiplied by $e^{i\zeta_{1}}$, and let $\Gamma =\sigma T_{1}+\varsigma_{1}$. If the frequency response is a steady state, $\Gamma^{*}=\sigma +\varsigma_{1}^{*}=0$, we can know $\varsigma_{1}^{*}=-\sigma$. The real part of the 1st mode’s solvability condition can be expressed as:

(30) $$\begin{array}{l} 2\omega_{1}\sigma B_{1}-{\overset{⌢}{q}}_{1}\cos\Gamma -\frac{1}{Q_{1}}(3B_{1}\bar{B_{1}}B_{1}(\int_{0}^{1}\phi_{1}{\phi^{\prime \prime }}_{1}^{3}dx+\int_{0}^{1}\phi_{1}\phi_{1}^{iv}{\phi^{\prime }}_{1}{}^{2}dx+\int_{0}^{1}4\phi_{1}{\phi^{\prime }}_{1}{\phi^{\prime \prime }}_{1}{\phi^{\prime \prime \prime }}_{1}dx)\\ +2B_{1}\bar{B_{2}}B_{2}(\int_{0}^{1}3{\phi^{\prime \prime }}_{1}{\phi^{\prime \prime }}_{2}^{2}\phi_{1}dx+\int_{0}^{1}\phi_{1}\phi_{1}^{iv}{\phi^{\prime }}_{2}{}^{2}dx+\int_{0}^{1}2\phi_{1}\phi_{2}^{iv}{\phi^{\prime }}_{1}{\phi^{\prime }}_{2}dx+\int_{0}^{1}4{\phi^{\prime }}_{1}{\phi^{\prime \prime }}_{2}{\phi^{\prime \prime \prime }}_{2}\phi_{1}dx\\ +\int_{0}^{1}4{\phi^{\prime }}_{2}{\phi^{\prime \prime }}_{2}{\phi^{\prime \prime \prime }}_{1}\phi_{1}dx+\int_{0}^{1}4{\phi^{\prime }}_{2}{\phi^{\prime \prime }}_{1}{\phi^{\prime \prime \prime }}_{2}\phi_{1}dx)\\ +2B_{1}\bar{B_{3}}B_{3}(\int_{0}^{1}3{\phi^{\prime \prime }}_{1}{\phi^{\prime \prime }}_{3}^{2}\phi_{1}dx+\int_{0}^{1}\phi_{1}^{iv}\phi_{1}{\phi^{\prime }}_{3}{}^{2}dx+\int_{0}^{1}2\phi_{1}\phi_{3}^{iv}{\phi^{\prime }}_{1}{\phi^{\prime }}_{3}dx+\int_{0}^{1}4{\phi^{\prime }}_{1}{\phi^{\prime \prime }}_{3}{\phi^{\prime \prime \prime }}_{3}\phi_{1}dx\\ +\int_{0}^{1}4{\phi^{\prime }}_{3}{\phi^{\prime \prime }}_{3}{\phi^{\prime \prime \prime }}_{1}\phi_{1}dx+\int_{0}^{1}4{\phi^{\prime }}_{3}{\phi^{\prime \prime }}_{1}{\phi^{\prime \prime \prime }}_{3}\phi_{1}dx))\\ -\frac{1}{2Q_{1}}(-4\omega_{1}^{2}B_{1}\bar{B_{1}}B_{1}(\int_{0}^{1}{\phi^{\prime \prime }}_{1}\phi_{1}(\int_{1}^{x}\int_{0}^{x}{\phi^{\prime }}_{1}{\phi^{\prime }}_{1}dxdx)dx+\int_{0}^{1}{\phi^{\prime }}_{1}\phi_{1}(\int_{1}^{x}\int_{0}^{x}{\phi^{\prime }}_{1}{\phi^{\prime }}_{1}dxdx{)}^{\prime }dx)\\ +4(-\omega_{1}^{2}-\omega_{2}^{2})B_{1}\bar{B_{2}}B_{2}(\int_{0}^{1}{\phi^{\prime \prime }}_{2}\phi_{1}(\int_{1}^{x}\int_{0}^{x}{\phi^{\prime }}_{1}{\phi^{\prime }}_{2}dxdx)dx)+\int_{0}^{1}{\phi^{\prime }}_{2}\phi_{1}(\int_{1}^{x}\int_{0}^{x}{\phi^{\prime }}_{1}{\phi^{\prime }}_{2}dxdx{)}^{\prime }dx)\\ +4(-\omega_{1}^{2}-\omega_{3}^{2})B_{1}\bar{B_{3}}B_{3}(\int_{0}^{1}{\phi^{\prime \prime }}_{3}\phi_{1}(\int_{1}^{x}\int_{0}^{x}{\phi^{\prime }}_{1}{\phi^{\prime }}_{3}dxdx)dx\\ +\int_{0}^{1}{\phi^{\prime }}_{3}\phi_{1}(\int_{1}^{x}\int_{0}^{x}{\phi^{\prime }}_{1}{\phi^{\prime }}_{3}dxdx{)}^{\prime }dx)) \end{array}$$

The imaginary part is:

(31) $$-\mu \omega_{1}B_{1}-{\overset{⌢}{q}}_{1}\sin\Gamma -C_{G}\frac{1}{e^{R\tau }}\int_{0}^{\tau }\omega_{1}B_{1}e^{R\tau }d\tau$$

**Figure 4:**
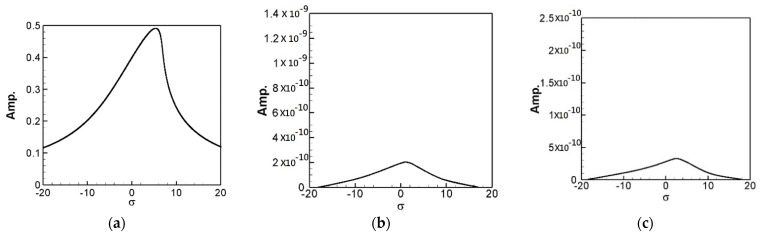
SES system fixed points plots of the 1st mode being excited. (**a**) 1st mode response, (**b**) 2nd mode response, and (**c**) 3rd mode response.

**Figure 5:**
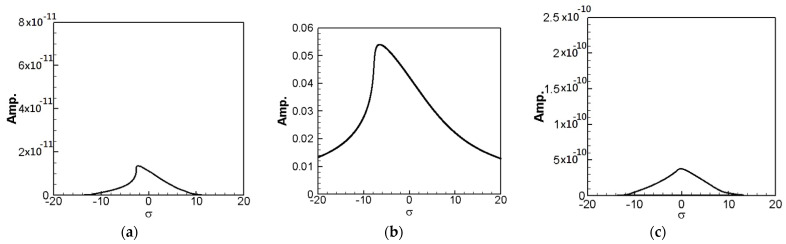
SES system fixed points plots of the 2nd mode being excited. (**a**) 1st mode response, (**b**) 2nd mode response, and (**c**) 3rd mode response.

**Figure 6:**
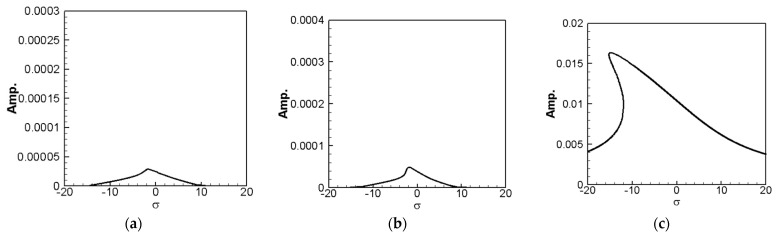
SES system fixed points plots of the 3rd mode being excited. (**a**) 1st mode response, (**b**) 2nd mode response, and (**c**) 3rd mode response.

In the same way, multiply the 2nd and 3rd modes’ secular terms by $e^{i\varsigma_{2}}$ and $e^{i\varsigma_{3}}$, respectively to obtain their solvability conditions. In order to eliminate the time correlated term (Γ), the squares of Equations (30) and (31) are added up. These solvability conditions can be solved numerically by using IMSL© subroutine—NEQNF nonlinear solver and the Levenberg-Marquardt algorithm. The response plot of amplitudes *B*_1_, *B*_2_, and *B*_3_ vs. the tuned frequency (σ) of this system can be drawn as fixed points plots. Figure 4, Figure 5 and Figure 6 are the frequency responses for the 1st, 2nd, and 3rd modes, respectively. The maximum amplitude happens near the linear natural frequency (σ = 0) for each mode. Those values are used for the perturbation method in the next section.

## 4. SES VEH Power Generation Benefit Analysis

**Figure 7:**
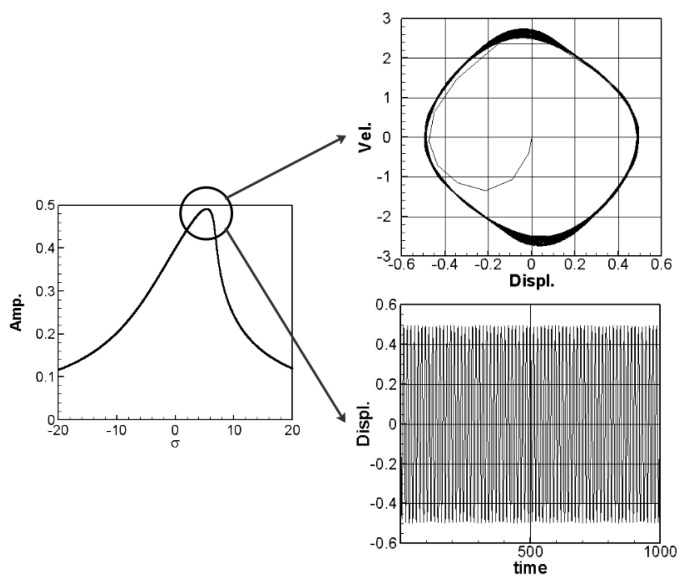
SES verification diagrams of fixed-point plots, phase plots, and time response plots of the 1st mode’s forced response.

**Figure 8:**
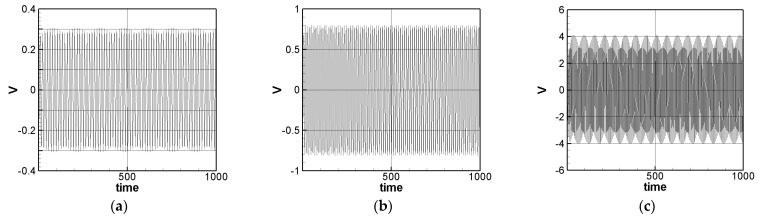
SES system theoretical voltage output (dimensionless). (**a**) 1st mode, (**b**) 2nd mode, and (**c**) 3rd mode.

This study uses the perturbation technique to analyze the power benefit of the nonlinear SES VEH system. The $\xi_{n}$ of this beam can be expressed as: $\xi_{n}={\bar{\xi }}_{n}+{\tilde{\xi }}_{n}$, where ${\bar{\xi }}_{n}$ is the equilibrium term and is obtained from Section 3.2 (the steady-state response *B*_n_), ${\tilde{\xi }}_{n}$ is the disturbance term. It is noted that the equilibrium term ${\bar{\xi }}_{n}$ is chosen as the maximum amplitude form the fixed points plot from Figure 4, Figure 5 and Figure 6 for each mode to show the best effect of the theoretical model. $W=\sum_{n=1}^{3}({\bar{\xi }}_{n}+{\tilde{\xi }}_{n})\phi_{n}$ is then substituted into Equation (17) and orthogonalized as:

$$\begin{array}{l} {\bar{\xi }}_{m}^{**}+{\tilde{\xi }}_{m}^{**}+{\bar{\xi }}_{m}\phi_{m}{}^{iv}+{\tilde{\xi }}_{m}\phi_{m}{}^{iv}+\mu {\bar{\xi }}_{m}^{*}+\mu {\tilde{\xi }}_{m}^{*}\\ +\frac{1}{Q_{m}}(\int_{0}^{1}\phi_{m}{\left({\bar{\xi }}_{m}+{\tilde{\xi }}_{m}\right)}^{3}{\phi^{\prime \prime }}_{m}{}^{3}dx+\int_{0}^{1}\phi_{m}({\bar{\xi }}_{m}+{\tilde{\xi }}_{m})\phi_{m}{}^{iv}\phi_{m}{\phi^{\prime }}_{m}{}^{2}dx\\ +4\int_{0}^{1}\phi_{m}{\left({\bar{\xi }}_{m}+{\tilde{\xi }}_{m}\right)}^{3}\phi_{m}{}^{\prime }\phi_{m}{}^{\prime \prime }\phi_{m}{}^{\prime \prime \prime }dx)+\frac{1}{2Q_{m}}(({\bar{\xi }}_{m}+{\tilde{\xi }}_{m})(({\bar{\xi }}_{m}+{\tilde{\xi }}_{m})**({\bar{\xi }}_{m}+{\tilde{\xi }}_{m})\\ +2({\bar{\xi }}_{m}+{\tilde{\xi }}_{m})*({\bar{\xi }}_{m}+{\tilde{\xi }}_{m})*\\ +({\bar{\xi }}_{m}+{\tilde{\xi }}_{m})({\bar{\xi }}_{m}+{\tilde{\xi }}_{m})**)(\int_{0}^{1}\phi_{m}\phi_{m}{}^{\prime \prime }\int_{1}^{x}\int_{0}^{x}{\phi^{\prime }}_{m}{}^{2}dxdx+\int_{0}^{1}\phi_{m}\phi_{m}{}^{\prime }(\int_{1}^{x}\int_{0}^{x}{\phi^{\prime }}_{m}{}^{2}dxdx)\prime )\\ -\frac{1}{Q_{m}}(\frac{\hat{k}\eta^{2}}{e^{R\tau }}({\bar{\xi }}_{m}+{\tilde{\xi }}_{m})\int_{0}^{1}\phi_{m}{\left(\int_{0}^{1}{\phi^{\prime \prime }}_{m}dx\right)}^{2}(\int_{0}^{\tau }{\left({\bar{\xi }}_{m}+{\tilde{\xi }}_{m}\right)}^{*}e^{R\tau }d\tau )dx) \end{array}$$

(32) $$+C_{G}\frac{1}{e^{R\tau }}\int_{0}^{\tau }{\left({\bar{\xi }}_{m}+{\tilde{\xi }}_{m}\right)}^{*}e^{R\tau }d\tau -\frac{1}{Q_{m}}\int_{0}^{l}\phi_{m}q_{m}dx=0$$

where *m* = 1–3. The solution of Equation (32) is carried out using the RK-4 method, and the response of voltage *v* and time *t* of the system is made as a time response of a nonlinear SES VEH system. Figure 7 gives an example of the mutual verification diagrams of fixed-point plots, phase plots, and time response plots of the 1st mode’s forced response, which prove the correctness of the theoretical model. We learned the amplitude of each mode through the fixed points plots in Section 3.2, and substituted it into the piezoelectric equation, and obtained the voltage and time of the 1st, 2nd, and 3rd mode by the RK-4 method (Figure 8). The results show that as the vibration frequency increases, the power generation volts and benefits also increase.

## 5. DES VEH Power Generation Benefit Analysis

### 5.1. DES Frequency Response

**Figure 9:**
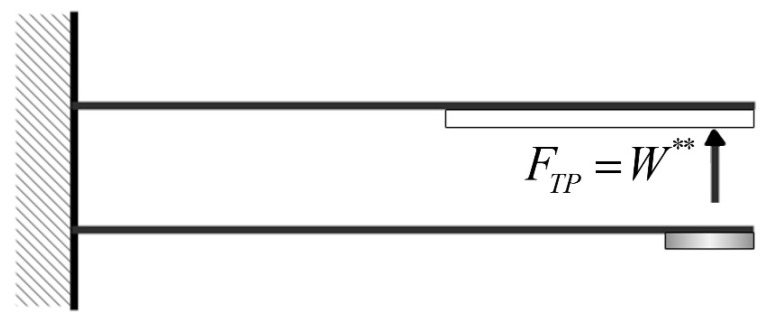
Schematic of the double elastic beam.

This study assumes that the piezo patch is placed at the free end of the beam. The flapping force on the piezo patch from the other elastic steel sheet can be expressed in the dimensionless form as $F_{TP}=W^{**}$, as shown in Figure 9.

Since $\xi =A_{P}e^{i\omega t}$, *F_TP_* = $-A_{P}\omega^{2}\sin\omega t\phi (x){|}_{x=1}^{}$. The concentrated load can be considered the external force of SES, in which *A_P_* is the amplitude of the other elastic steel on the tip of the beam (*x* = 1), which can be obtained by MOMS from Section 3.2. Finally, the equation of motion for the nonlinear DES system using the flapping external force of the piezo patch is expressed as follows:

(33) $$\begin{array}{l} \;\frac{\partial^{2}W_{1}}{\partial T_{0}{}^{2}}+W_{1}{}^{iv}=-2\;\frac{\partial^{2}W_{0}}{\partial T_{0}\partial T_{1}}-\mu \frac{\partial W_{0}}{\partial T_{0}}-{W^{\prime \prime }}_{0}{}^{\;3}-W_{0}^{\mathrm{iv}}{W^{\prime }}_{0}{}^{\;2}-4{W^{\prime }}_{0}\;{W^{\prime \prime \prime }}_{0}\;{W^{\prime \prime }}_{0}\;\\ -\frac{1}{2}({W^{\prime }}_{0}\int_{1}^{x}{\left(\int_{0}^{x}{W^{\prime }}_{0}{}^{2}dx\right)}^{**}dx{)}^{\prime }-C_{G}\frac{1}{e^{R\tau }}\int_{0}^{\tau }(\frac{\partial W_{0}}{\partial T_{0}})e^{R\tau }d\tau \\ +\frac{\hat{k}\eta^{2}}{e^{R\tau }}\int_{a}^{b}{W^{\prime \prime }}_{0}dx\int_{0}^{\tau }(\int_{a}^{b}(\frac{\partial^{3}W_{0}}{\partial T_{0}\partial x^{2}})dxe^{R\tau })d\tau +q+W^{**} \end{array}$$

By using orthogonal properties, the 1st mode’s secular terms can be found and the solvability condition is written as:

(34) $$\begin{array}{l} -2(i\omega_{1}B_{1}e^{i\zeta_{1}}e^{i\omega_{1}T_{0}}-\omega_{1}\varsigma_{1}^{*}B_{1}e^{-i\zeta_{1}}e^{i\omega_{1}T_{0}})-\mu (i\omega_{1}B_{1}e^{-i\varsigma_{1}}e^{i\omega_{1}T_{0}})+{\overset{⌢}{q}}_{1}e^{i\omega_{1}T_{0}}e^{i\sigma T_{1}}\\ -\frac{1}{Q_{1}}(3B_{1}\bar{B_{1}}B_{1}e^{-i\varsigma_{1}}e^{i\omega_{1}T_{0}}(\int_{0}^{1}\phi_{1}{\phi^{\prime \prime }}_{1}^{3}dx+\int_{0}^{1}\phi_{1}\phi_{1}^{iv}{\phi^{\prime }}_{1}{}^{2}dx+\int_{0}^{1}4\phi_{1}{\phi^{\prime }}_{1}{\phi^{\prime \prime }}_{1}{\phi^{\prime \prime \prime }}_{1}dx)\\ +2B_{1}\bar{B_{2}}B_{2}e^{-i\varsigma_{1}}e^{i\omega_{1}T_{0}}(\int_{0}^{1}3{\phi^{\prime \prime }}_{1}{\phi^{\prime \prime }}_{2}^{2}\phi_{1}dx+\int_{0}^{1}\phi_{1}\phi_{1}^{iv}{\phi^{\prime }}_{2}{}^{2}dx+\int_{0}^{1}2\phi_{1}\phi_{2}^{iv}{\phi^{\prime }}_{1}{\phi^{\prime }}_{2}dx\\ +\int_{0}^{1}4{\phi^{\prime }}_{1}{\phi^{\prime \prime }}_{2}{\phi^{\prime \prime \prime }}_{2}\phi_{1}dx+\int_{0}^{1}4{\phi^{\prime }}_{2}{\phi^{\prime \prime }}_{2}{\phi^{\prime \prime \prime }}_{1}\phi_{1}dx+\int_{0}^{1}4{\phi^{\prime }}_{2}{\phi^{\prime \prime }}_{1}{\phi^{\prime \prime \prime }}_{2}\phi_{1}dx)\\ +2B_{1}\bar{B_{3}}B_{3}e^{-i\varsigma_{1}}e^{i\omega_{1}T_{0}}(\int_{0}^{1}3{\phi^{\prime \prime }}_{1}{\phi^{\prime \prime }}_{3}^{2}\phi_{1}dx+\int_{0}^{1}\phi_{1}^{iv}\phi_{1}{\phi^{\prime }}_{3}{}^{2}dx+\int_{0}^{1}2\phi_{1}\phi_{3}^{iv}{\phi^{\prime }}_{1}{\phi^{\prime }}_{3}dx\\ +\int_{0}^{1}4{\phi^{\prime }}_{1}{\phi^{\prime \prime }}_{3}{\phi^{\prime \prime \prime }}_{3}\phi_{1}dx+\int_{0}^{1}4{\phi^{\prime }}_{3}{\phi^{\prime \prime }}_{3}{\phi^{\prime \prime \prime }}_{1}\phi_{1}dx+\int_{0}^{1}4{\phi^{\prime }}_{3}{\phi^{\prime \prime }}_{1}{\phi^{\prime \prime \prime }}_{3}\phi_{1}dx))\\ -\frac{1}{2Q_{1}}(-4\omega_{1}^{2}B_{1}\bar{B_{1}}B_{1}e^{-i\varsigma_{1}}e^{i\omega_{1}T_{0}}(\int_{0}^{1}{\phi^{\prime \prime }}_{1}\phi_{1}(\int_{1}^{x}\int_{0}^{x}{\phi^{\prime }}_{1}{\phi^{\prime }}_{1}dxdx)dx\\ +\int_{0}^{1}{\phi^{\prime }}_{1}\phi_{1}(\int_{1}^{x}\int_{0}^{x}{\phi^{\prime }}_{1}{\phi^{\prime }}_{1}dxdx\;)\;\prime dx)\\ +4(-\omega_{1}^{2}-\omega_{2}^{2})B_{1}\bar{B_{2}}B_{2}e^{-i\varsigma_{1}}e^{i\omega_{1}T_{0}}(\int_{0}^{1}{\phi^{\prime \prime }}_{2}\phi_{1}(\int_{1}^{x}\int_{0}^{x}{\phi^{\prime }}_{1}{\phi^{\prime }}_{2}dxdx)dx\\ +\int_{0}^{1}{\phi^{\prime }}_{2}\phi_{1}(\int_{1}^{x}\int_{0}^{x}{\phi^{\prime }}_{1}{\phi^{\prime }}_{2}dxdx\;)\;\prime dx)\\ +4(-\omega_{1}^{2}-\omega_{3}^{2})B_{1}\bar{B_{3}}B_{3}e^{-i\varsigma_{1}}e^{i\omega_{1}T_{0}}(\int_{0}^{1}{\phi^{\prime \prime }}_{3}\phi_{1}(\int_{1}^{x}\int_{0}^{x}{\phi^{\prime }}_{1}{\phi^{\prime }}_{3}dxdx)dx\\ +\int_{0}^{1}{\phi^{\prime }}_{3}\phi_{1}(\int_{1}^{x}\int_{0}^{x}{\phi^{\prime }}_{1}{\phi^{\prime }}_{3}dxdx\;)\;\prime dx))-C_{G}\frac{1}{e^{R\tau }}\int_{0}^{\tau }(i\omega_{1})(B_{1}e^{-i\zeta_{1}}e^{i\omega_{1}T_{0}})e^{R\tau }d\tau \\ +\frac{1}{Q_{1}}(-\omega_{1}^{2}B_{1}e^{-i\zeta_{1}}e^{i\omega_{1}T_{0}})\int_{0}^{l_{p}}\phi_{1}{\phi_{1}(x)|}_{x=1}dx=0 \end{array}$$

Similarly, the 2nd and 3rd modes’ solvability conditions can be found. The fixed points plot and the frequency response for each mode can be made. The fixed-point plots are not shown for the sake of simplicity.

### 5.2. DES VEH Power Generation Benefit Analysis

Substituting the slapping force (from Section 5.1) into Equation (32), we can obtain the nonlinear DES VEH equation as:

(35) $$\begin{array}{l} {\bar{\xi }}_{m}^{**}+{\tilde{\xi }}_{m}^{**}+{\bar{\xi }}_{m}\phi_{m}{}^{iv}+{\tilde{\xi }}_{m}\phi_{m}{}^{iv}+\mu {\bar{\xi }}_{m}^{*}+\mu {\tilde{\xi }}_{m}^{*}\\ +\frac{1}{Q_{m}}(\int_{0}^{1}\phi_{m}{\left({\bar{\xi }}_{m}+{\tilde{\xi }}_{m}\right)}^{3}{\phi^{\prime \prime }}_{m}{}^{3}dx+\int_{0}^{1}\phi_{m}({\bar{\xi }}_{m}+{\tilde{\xi }}_{m})\phi_{m}{}^{iv}\phi_{m}{\phi^{\prime }}_{m}{}^{2}dx\\ +4\int_{0}^{1}\phi_{m}{\left({\bar{\xi }}_{m}+{\tilde{\xi }}_{m}\right)}^{3}\phi_{m}{}^{\prime }\phi_{m}{}^{\prime \prime }\phi_{m}{}^{\prime \prime \prime }dx)+\frac{1}{2Q_{m}}(({\bar{\xi }}_{m}+{\tilde{\xi }}_{m})(({\bar{\xi }}_{m}+{\tilde{\xi }}_{m}{)}^{**}({\bar{\xi }}_{m}+{\tilde{\xi }}_{m})\\ +2({\bar{\xi }}_{m}+{\tilde{\xi }}_{m}{)}^{*}({\bar{\xi }}_{m}+{\tilde{\xi }}_{m}{)}^{*}+({\bar{\xi }}_{m}+{\tilde{\xi }}_{m})({\bar{\xi }}_{m}+{\tilde{\xi }}_{m}{)}^{**}))(\int_{0}^{1}\phi_{m}\phi_{m}{}^{\prime \prime }\int_{1}^{x}\int_{0}^{x}{\phi^{\prime }}_{m}{}^{2}dxdx\\ +\int_{0}^{1}\phi_{m}\phi_{m}{}^{\prime }(\int_{1}^{x}\int_{0}^{x}{\phi^{\prime }}_{m}{}^{2}dxdx{)}^{\prime })-\frac{1}{Q_{m}}(\frac{\hat{k}\eta^{2}}{e^{R\tau }}({\bar{\xi }}_{m}+{\tilde{\xi }}_{m})\int_{0}^{1}\phi_{m}{\left(\int_{0}^{1}{\phi^{\prime \prime }}_{m}dx\right)}^{2}\\ (\int_{0}^{\tau }{\left({\bar{\xi }}_{m}+{\tilde{\xi }}_{m}\right)}^{*}e^{R\tau }d\tau )dx)+C_{G}\frac{1}{e^{R\tau }}\int_{0}^{\tau }{\left({\bar{\xi }}_{m}+{\tilde{\xi }}_{m}\right)}^{*}e^{R\tau }d\tau \\ -\frac{1}{Q_{m}}\int_{0}^{1}\phi_{m}q_{m}dx+\frac{1}{Q_{m}}({\bar{\xi }}_{m}+{\tilde{\xi }}_{m}{)}^{**}\int_{0}^{l_{p}}\phi_{m}{\phi_{m}(x)|}_{x=l_{p}}dx=0 \end{array}$$

**Figure 10:**
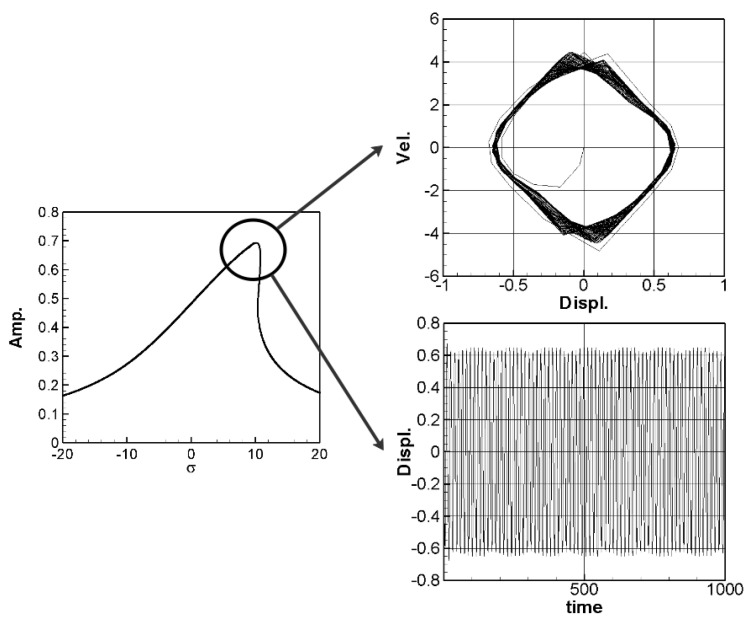
DES verification diagrams of fixed-point plots, phase plots, and time response plots of the 1st mode’s forced response.

**Figure 11:**
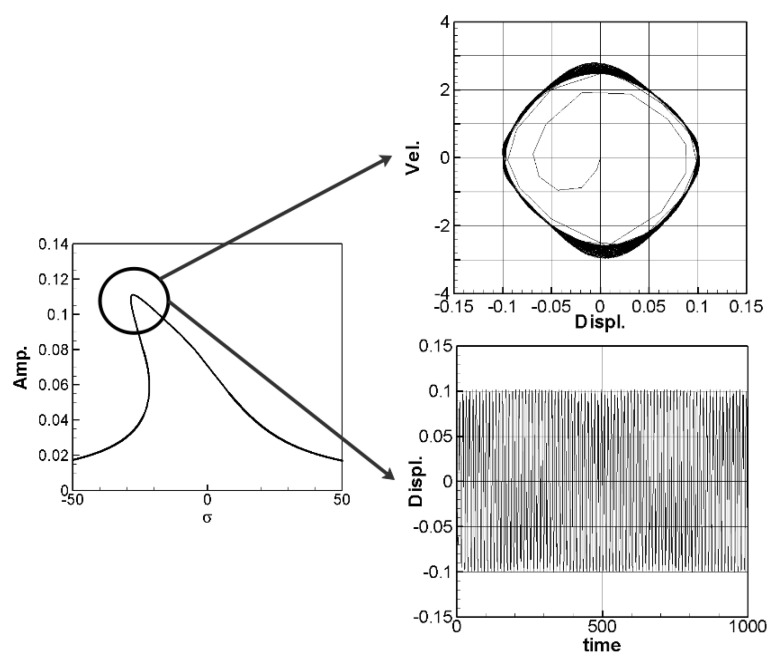
DES verification diagrams of fixed-point plots, phase plots, and time response plots of the 2nd mode’s forced response.

**Figure 12:**
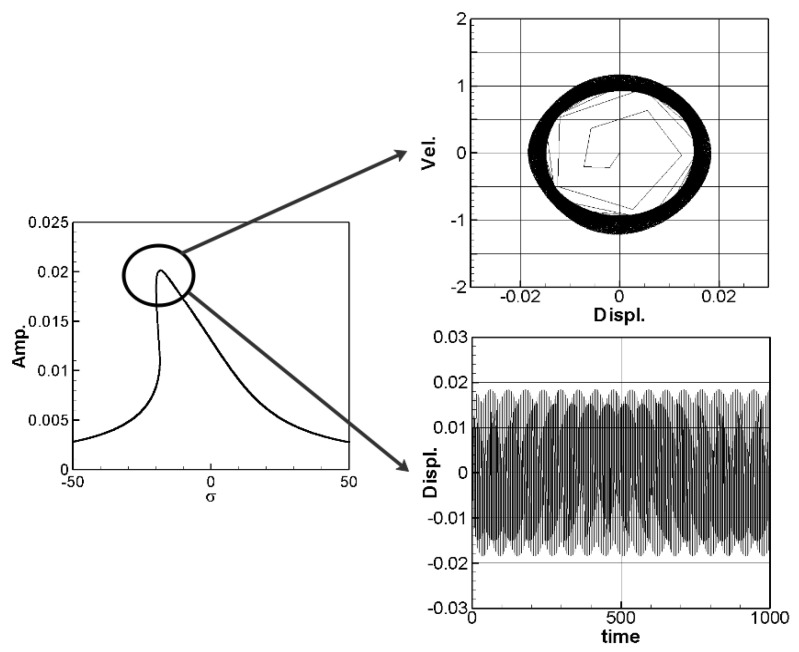
DES verification diagrams of fixed-point plots, phase plots, and time response plots of the 3rd mode’s forced response.

**Figure 13:**
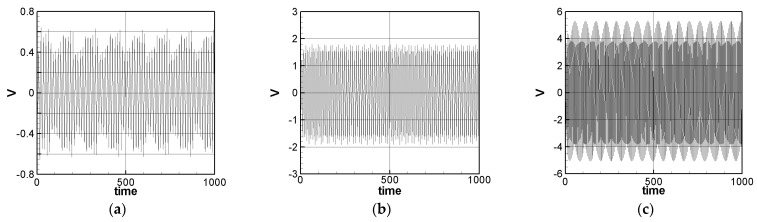
DES VEH system theoretical voltage output (dimensionless). (**a**) 1st mode, (**b**) 2nd mode, and (**c**) 3rd mode.

The solution of Equation (35) is carried out using the RK-4 method, and the response of voltage *v* and time *t* of the system is made as a time response of a nonlinear DES VEH system. Figure 10, Figure 11 and Figure 12 are the mutual verification diagrams of the fixed-point plots, phase plots, and time response plots of the 1st, 2nd, and 3rd modes’ forced response, which prove the correctness of the theoretical model. In the same way, the perturbation equation of the 1st, 2nd, and 3rd modes can be obtained by substituting the amplitude from the fixed points plots (in Section 3.2) into the piezoelectric equation. The voltage output can be obtained by using the RK-4 method and the results are shown in Figure 13. The results show that as the vibration frequency increases, the power generation volts and benefits also increase.

## 6. Experiment Setup

**Figure 14:**
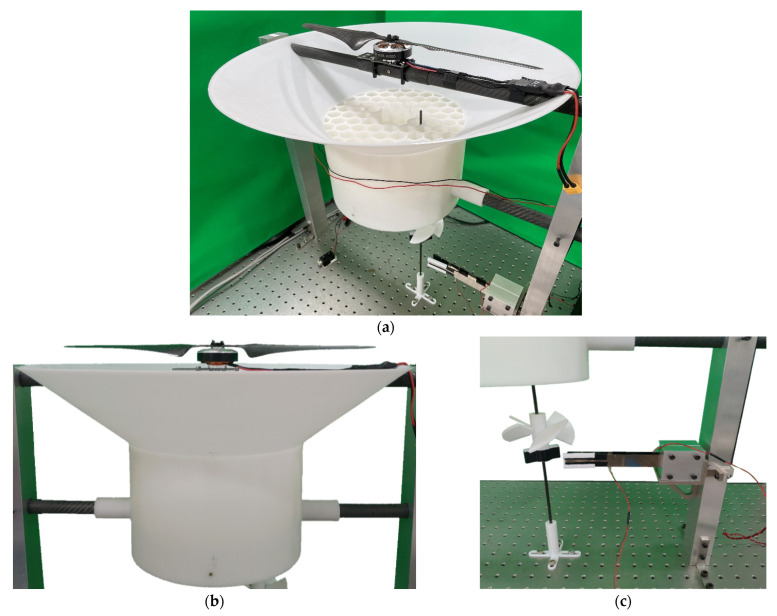
The assembly of the wind driven magneto-electric vibration energy harvester system. (**a**) brushless motor with a two-bladed rotor, (**b**) wind collecting cover, (**c**) windmill, and the magneto-electric VEH system.

We construct a simple experiment with the schematic diagram of the 3D theoretical model shown in Figure 1. This mechanism is composed of three parts, namely a brushless motor with a two-bladed rotor, a wind collecting cover, and the energy harvesting system. The design principle of the energy harvesting system in this study is to collect the airflow under the rotor and turn the windmill to drive the magnets of the system; then, the magnetic field is periodically transformed to form a repulsive force that excites the elastic steel, and the two elastic steels slap and vibrate each other, and generate electrical energy through the PZT. This study only discusses the situation of windmill blades at a pitch angle of 45°. The complete experimental setup is shown in Figure 14. Piezoelectricity is the electricity generated by piezo element by an effect known as the piezoelectric effect. It is the ability of certain materials to generate an AC (alternating current) voltage when subjected to mechanical stress or vibration, or to vibrate when subjected to an AC voltage, or both. If we take the example of PZT, the maximum current can vary from micro-Amperes to several Amperes, and the voltage generated in 1–100 V, depending upon the size of *PZT*. The experimental Piezo patch is the Bi-morph Type PZT (produced by Superex Technology^TM^). This PZT is one of the best of Superex Technology’s company. The dimension of the PZT is 60 mm × 20 mm. The distance between the two elastic beams is 1/13 of the elastic beam. This distance was chosen randomly, to ensure that the elastic beams can flap freely and slap each other. The purpose of this research is to prove that the concept of the DES VEH has better power generation than the traditional SES VEH system. The distance between the two elastic beams selected in this study has shown its capability in generating power. The other different dimensions of the PZT and the different distance between the two elastic beams may result in different power generation. This will be recommended for future work in this study.

As shown in Figure 14a,c, the windmill is installed directly under the middle of the wind collector. Most of the airflow enters the windmill. The only external force exerted on the beam is the repulsive force between the magnets. The beams are installed parallel to the airflow and keep a little distance from the middle of the air collector. This will allow the beams to withstand less airflow impact and less damage to the PZT sensor. This will also avoid the random vibration caused by external loads other than the magnetic force. In this study, a honeycomb wind collector was designed, as shown in Figure 14a, to collect the airflow from the downstream of the rotor blades. This honeycomb structure also rectifies the airflow to make it move smoothly, thereby exciting the windmill. The rotor speed used in the experimental setup was set to a maximum of 2000 RPM which almost reaches the maximum speed as the same type of rotor installed on a multi-axes drone. Since the wind collector was installed and the beams are installed parallel to the airflow and keep a little distance from the middle of the air collector. This somehow reduces the direct impact of the airflow on the beams and the magnet. No changes in the beams and the magnet were observed during the entire experiment. The only factor that excites the beams was the magnetic repulsive force as designed in this study.

### 6.1. Natural Frequency and Internal Resistance

We use impact hammer, accelerometer, and fast Fourier transform (from the IMC© system) to measure the first three natural frequencies of this system, which are 3.98 Hz, 19.53 Hz, and 54.35 Hz. From Equation (22), substituting the length of the beam and the mass ratio of the magnet to the beam, the first three eigen values can be obtained as: 2.1264, 4.7142, and 7.8590. The theoretical natural frequency of the first three modes can be calculated as: 3.980 Hz, 19.562 Hz, and 54.367 Hz. These values are consistent with the frequencies from the experimental analysis. Before analyzing the power of the wind-driven magneto-electric energy harvester system, it is necessary to add resistance to the system as the system load. In order to obtain the best power of the system, the closer the additional resistance value is to the internal resistance of the system, the best electrical power can be obtained. The following describes the estimation of internal resistance. First, the electric power equation is as follows:

(36) $$P=\bar{I}V={\bar{I}}^{2}R=\frac{V^{2}}{R}$$

where *P* is power, $\bar{I}$ denotes current, *V* represents voltage, and *R* is resistance.

We use Thevenin’s Theorem to find the internal resistance. Thevenin’s Theorem gives,

(37) $$V_{L}=\frac{R_{L}}{R_{T}+R_{L}}V_{T}$$

where $R_{T}$ is internal resistance, $R_{L}$ denotes the loading resistance, $V_{T}$ is the open circuit voltage, and $V_{L}$ is the loading voltage. From Equations (36) and (37) we can obtain:

(38) $$P=\frac{V_{L}^{2}}{R_{L}}=\frac{\frac{R_{L}^{2}}{{\left(R_{T}+R_{L}\right)}^{2}}V_{T}^{2}}{R_{L}}=\frac{R_{L}V_{T}^{2}}{{\left(R_{T}-R_{L}\right)}^{2}+4R_{T}R_{L}}=\frac{V_{T}^{2}}{\frac{{\left(R_{T}-R_{L}\right)}^{2}}{R_{L}}+4R_{T}}$$

When the loading resistance is equal to the internal resistance of the system, the best electric power will be obtained.

**Figure 15:**
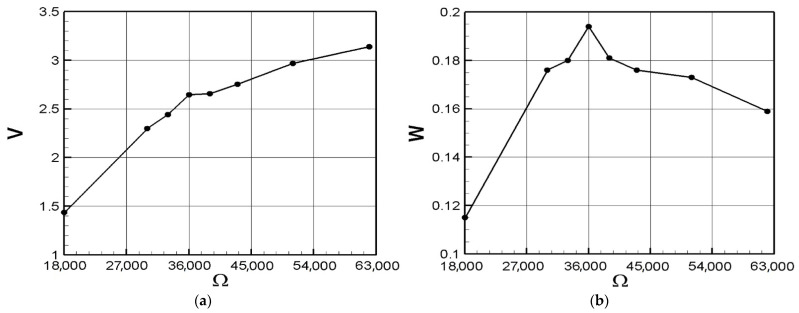
Diagram of ohm-volt and ohm-power, (**a**) ohm-volt diagram, and (**b**) ohm-power diagram.

We used the IMC^©^ system to obtain the open-circuit voltage of the system and used low-pass filtering to filter out high-frequency noise, and then use the Butterworth filter to obtain the voltage. Excite the 1st mode of the VEH system, and its output voltage is shown in Table 1. Take the root mean square and the average voltage is 4.3454 volts. In the next step, we choose 18 K ohm as the load of the system, and the average value of the output voltage is 1.4377 volts. Substituting this result into Equation (38), the theoretical internal resistance value can be calculated as 36,404 ohms (about 36 K ohms). We also tested the load resistance from 30 K to 62 K and obtained the output voltage and power as shown in Table 1. Through Table 1, the ohm-volt diagram and the ohm-power diagram can be made as shown in Figure 15. It can be observed that when the load resistance is 36 K ohms, the highest output power is 0.194 mW. Therefore, in the following Sections, when measuring the power generation efficiency of the system, 36 K ohms will be used as the experimental load resistance.

### 6.2. SES and DES System Output Voltage

**Figure 16:**
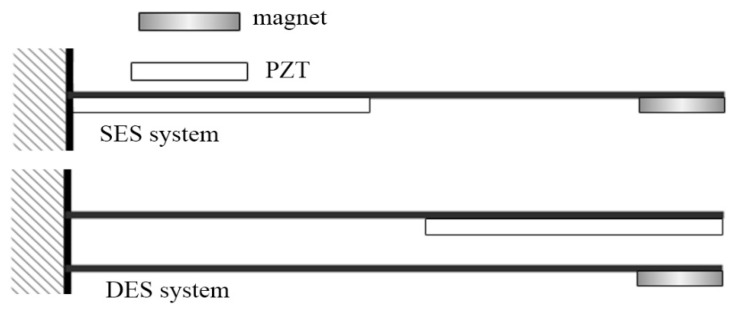
Schematic of SES and DES systems.

In the experiment, we used an inverter motor to drive the RPM of the windmill. When the 1st mode, 2nd mode, and 3rd mode were excited, the windmill RPM was about 120, 480, and 1650. We tested two VEH systems as shown in Figure 16. Figure 16 shows the traditional SES VEH with the PZT patch placed at the root of the elastic steel, and the DES VEH system with the PZT patch placed on the free end of one of the elastic steels and place the magnet on the free end of the other elastic steel. The PZT patch is deformed and slapped to generate a voltage in the DES system. The experimental results of the voltage output of the SES and DES systems are shown in Table 2 and Table 3, respectively.

**Figure 17:**
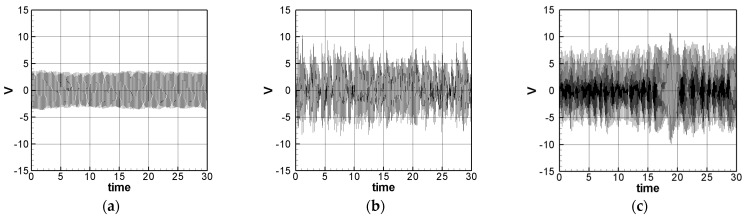
SES system experimental voltage output (with dimension (volt)). (**a**) 1st mode, (**b**) 2nd mode, and (**c**) 3rd mode.

**Figure 18:**
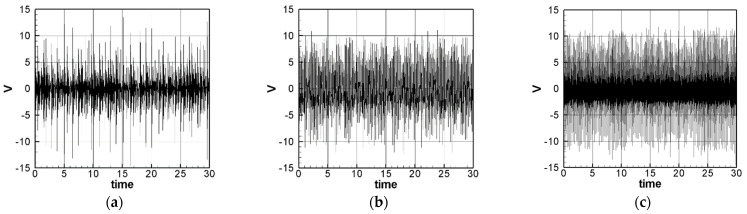
DES system experimental voltage output (with dimension (volt)). (**a**) 1st mode, (**b**) 2nd mode, and (**c**) 3rd mode.

We have observed in the experiments that whether it is SES or DES system, the higher the mode, the higher the volts of power generation, which is consistent with the trend of the theoretical prediction (Figure 8 and Figure 13). In addition, it is also found on the SES system that if the vibration frequency is too large, the repulsive force of the magnet and the restoring force of the elastic steel cannot be fully matched, resulting in the elastic steel sometimes having a large swing amplitude, sometimes it only vibrates at a small amplitude at the original equilibrium position, which is repeated iteratively. Figure 17 shows the voltage output diagram of the first three modes of the SES system. It can be observed that the frequency of the 3rd mode of the SES system is too fast at 15 to 20 s, resulting in the elastic steel vibration being unable to match the frequency generated by the magnet repulsion. The elastic steel has irregular amplitude. This phenomenon is unfavorable for power generation. In the experiment of the DES system (see Figure 18), in addition to the original repulsion force and the impact of the slap force, the elastic steel has a larger amplitude Moreover, because of the impact of the slap force and at high frequencies, the repulsive force of the magnet cannot match the elastic force of the steel, just like the SES system. As a result, sometimes the voltage output is not good. Therefore, if the repulsive force of the magnet can be effectively adjusted or the slap force can be reduced, in theory, a better slap situation can be achieved. Therefore, the DES system can meet the theoretical prediction.

## 7. Theoretical and Experimental Verification

This Section will verify the theoretical and experimental results of the SES and DES system. We took the beam mass, length, magnet mass, etc., used in the experiment, and put these parameters into the theoretical functions, and got the dimensional output voltages. The root mean squares (RMS) of theoretical output voltages of the SES and DES system are detailed in Table 4. It can be observed that the voltages of either the SES or DES system increase with an increase in the modes, and the comparison with the experimental results also has the same trend. Through experiments, it is found that the SES system or the DES system is relatively stable in the first two modes. When the third mode is excited, the frequency is too fast, resulting in an irregular vibration of the elastic steel. Especially when the 3rd mode is excited in the DES system, the impact of the flapping force destroys the periodic vibration of the elastic steel. The repulsive force of the magnet and the restoring force of the elastic steel on both of the SES and DES systems cannot be fully matched, resulting in the elastic steel sometimes having a large swing amplitude, sometimes it only vibrates at a small amplitude at the original equilibrium position, which is repeated iteratively. This phenomenon is unfavorable for power generation. In the experiment of the DES system, in addition to the original repulsion force and the impact of the slap force, the elastic steel has a larger amplitude. Moreover, because of the impact of the slap force and at high frequencies, the repulsive force of the magnet cannot match the elastic force of the steel, just like the SES system. As a result, sometimes the voltage output is not good. Due to these factors, the experiments of the SES system or the DES system in the 3rd mode cannot agree with the theoretical results. In future engineering, if the repulsive force of a magnet can be designed and adjusted to match the flapping force of the elastic steel so that it can be more idealized, we believe it will have a better effect.

**Figure 19:**
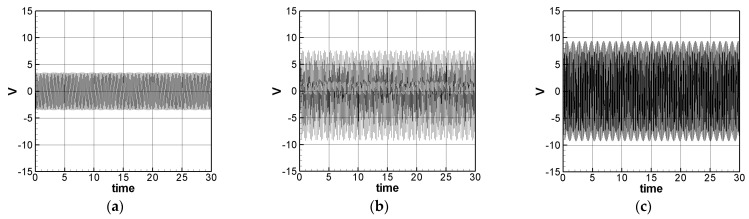
SES system theoretical voltage output (with dimension (volt)). (**a**) 1st mode, (**b**) 2nd mode, and (**c**) 3rd mode.

**Figure 20:**
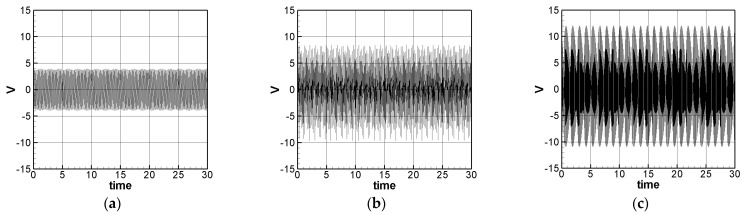
DES system theoretical voltage output (with dimension (volt)). (**a**) 1st mode, (**b**) 2nd mode, and (**c**) 3rd mode.

Table 2 shows the experimental results of the first three modes of the SES system. The first three modes root mean square (RMS) voltage is 2.1351, 3.1809, and 3.3836 volts, respectively. The ratio is 1:1.4898:1.5848. The theoretically estimated SES and DES system’s 1st, 2nd and 3rd mode’s voltage outputs with dimension are shown in Figure 19 and Figure 20. Figure 19 is for the SES system and Figure 20 is for the DES system, respectively. The theoretical root mean square (RMS) output voltages with dimension are shown in Table 4.

From Table 4, the root mean square voltages for the first three modes of the SES system are 2.2660, 3.4741, and 6.0436 respectively. The ratio is 1:1.5331:2.6671. According to the experimental results, it can be shown that the output voltage agrees with the theoretical prediction very well. The 1st mode output voltage relative error is 6.1308%, the 2nd mode output voltage relative error is 9.2175%, and the 3rd mode voltage relative error is 78.6145%. Then, compare the voltage ratio of the 1st and 2nd modes. The experimental voltage ratio of the first two modes is 1:1.4898, and the theoretical voltage ratio is 1:1.5331; the relative error of the ratio of the two modes is about 2.9064%. It is confirmed that the experimental values of the first two modes conform to the theoretical predictions.

Similarly, for the DES system, the first three modes of the experimental RMS voltage are 2.2824, 3.4469, and 3.5179 volts, respectively. The ratio is 1:1.5102:1.5413. The theoretically estimated DES system’s voltages are 2.4484, 3.7988, and 6.7436, respectively. The ratio is 1:1.5515:2.7543. The 1st mode output voltage relative error is 7.2730%, the 2nd mode output voltage relative error is 10.2092%, and the 3rd mode voltage relative error is 91.6939%. The experimental voltage ratio of the first two modes is 1:1.5102, and the theoretical voltage ratio is 1:1.5515; the relative error of the ratio of the two modes is about 2.7347%. It is confirmed that the experimental values of the first two modes conform to the theoretical predictions.

The objective of this work is to propose a DES VEH system and to show that this novel concept can provide a better power generation than the traditional SES VEH system. This study proves its characteristics theoretically. This research just carried out a simple experimental setup to demonstrate the working principle of the theoretical model. Future work may suggest more complex and precise experiments. However, compared with the absolute values of the theoretical and experimental voltage outputs, for the SES system, the 1st mode output voltage relative error is 6.1308%, the 2nd mode output voltage relative error is 9.2175%, and the 3rd mode voltage relative error is 78.6145%. For the DES system, the 1st mode output voltage relative error is 7.2730%, the 2nd mode output voltage relative error is 10.2092%, and the 3rd mode voltage relative error is 91.6939%. The errors of the first two modes are almost less 10% and can be considered acceptable for engineering applications. The 3rd mode is difficult to achieve its ideal flapping condition and it is hard to match the magnetic repulsive force as mentioned in this work. This may require future improvements. It can be seen that the theories and experiments of the first two modes of the SES system and the DES system can be mutually verified. In future engineering applications, proper adjustment of the repulsive force of the magnet will reduce the occurrence of irregular vibration in the third mode of the SES and DES system, in order to obtain a higher voltage output.

## 8. Conclusions

This research is based on the main structure of a fixed-free nonlinear elastic beam with a tip mass. The frequency response and amplitude are analyzed by the MOMS method and fixed points plots. The SES and DES VEH systems are excited by wind and magnetic force to convert the vibration energy to electric power. Through experiments to verify the correctness of the theory of this study. The nonlinear beam model also supports the results from the experiments of the double elastic steel energy harvesting system. The proposed DES system has better power generation than the traditional single elastic steel system, which was used in refs. [15,16]. The use of wind and magnetic force applied on the DES system also has a wider range of practical applications than a simple external load from a shaker, which was used in refs. [17,18,19]. The novel concept and practical application have been proved feasible in this work. The conclusions of this research are as follows:

1. Regardless of theory or experiment, the voltage outputs of the SES or DES system increase as the excitation modes increase and the voltage output of the DES system in each mode is also greater than that of the SES system.
2. The experimental voltage values of the SES and DES systems in the first and second modes agree with theoretical predictions. The reason for the large difference between the theoretical value and the experimental value of the 3rd mode is due to the irregular motion of the third mode. The frequency generated by the repulsive force of the magnet driven by the rotation of the windmill cannot be matched with the elastic force of the elastic steel, resulting in the 3rd mode not being able to produce the same voltage output as the theoretical value.
3. Regardless of whether it is an SES system or a DES system, the theoretical prediction in each mode is greater than the experimental result. According to the theoretical estimate, the voltage output is the 3rd mode > the 2nd mode > the 1st mode. The voltage output effect of the DES system is also better than that of the SES system.

In summary, we recommend using the DES energy harvesting system. However, when the 3rd mode is excited, the periodicity of the elastic steel is destroyed. In future engineering applications, if the repulsive force of the magnet can be adjusted to control the slap force on the elastic steel so that it can maintain a periodic state after slap, which will enable the third mode of the DES system to approach the theoretical condition. In the future, we hope that through this piezoelectric energy harvesting system driven by wind and magnetic force, we can collect the downward airflow generated by helicopters and multi-axis unmanned rotorcrafts and recycle this energy to maximize the effectiveness of DES VEH.

## Figures and Tables

**Table 1:** Experimental measurement of system resistance.

Resistance (ohm)	Voltage (V)						
	A	B	C	D	E	Average (V)	mW
18,000	1.4340	1.4284	1.4706	1.4326	1.4232	1.4377	0.1148
30,000	2.0682	2.2663	2.2344	2.4732	2.4556	2.2995	0.1763
33,000	2.2042	2.3711	2.4706	2.5141	2.6418	2.4404	0.1805
36,000	2.5687	2.6546	2.6215	2.7136	2.6590	2.6435	0.1941
39,000	2.6012	2.6111	2.7039	2.6727	2.6950	2.6568	0.1810
43,000	2.6584	2.8030	2.8323	2.7838	2.6848	2.7525	0.1762
51,000	3.0149	2.9188	2.9670	3.0128	2.9255	2.9678	0.1727
Open circuit (V)	4.5053	4.1695	4.2287	4.4984	4.3251	4.3454	

**Table 2:** Experimental results of the voltage and power of the SES system.

Case	SES Mode 1			SES Mode 2			SES Mode 3		
	Voltage (V)	mW	Max V	Voltage (V)	mW	Max V	Voltage (V)	mW	Max V
1	2.0563	0.1174	3.8102	3.2891	0.3005	10.3193	3.3664	0.3148	13.6078
2	2.0993	0.1224	3.8217	3.0991	0.2668	9.5380	3.2767	0.2982	10.3314
3	2.1410	0.1273	3.8929	3.2660	0.2963	11.2396	3.2085	0.2860	11.3319
4	2.1901	0.1332	3.7929	3.1179	0.2701	10.5006	3.5251	0.3452	13.3956
5	2.1886	0.1331	3.7948	3.1323	0.2725	9.3771	3.5416	0.3484	13.6456
Ave.	2.1351	0.1267	3.8225	3.1809	0.2812	10.1949	3.3836	0.3185	12.4625

**Table 3:** Experimental results of the voltage and power of the DES system.

Case	DES Mode 1			DES Mode 2			DES Mode 3		
	Voltage (V)	mW	Max V	Voltage (V)	mW	Max V	Voltage (V)	mW	Max V
1	2.2058	0.1352	14.11527	3.5632	0.3527	14.46878	3.4834	0.3371	14.9354
2	2.4851	0.1715	14.69722	3.2502	0.2934	13.64842	3.5404	0.3482	14.2494
3	2.3477	0.1531	14.05054	3.3983	0.3208	14.23503	3.3271	0.3075	14.5385
4	2.2659	0.1426	13.8839	3.7611	0.3929	14.05283	3.6828	0.3767	14.9435
5	2.1075	0.1234	13.99478	3.2616	0.2955	13.83062	3.556	0.3512	14.4558
Ave.	2.2824	0.1452	14.1483	3.4469	0.3311	14.0471	3.5179	0.3441	14.6245

**Table 4:** SES and DES system theoretical output voltage (with dimension (volt)).

		SES	DES
RMS voltage (V)	Mode1	2.2660	2.4484
	Mode2	3.4741	3.7988
	Mode3	6.0436	6.7436

## Data Availability

Not applicable.
